# C. difficile Infection Complicated by a Large Pleural Effusion

**DOI:** 10.7759/cureus.77460

**Published:** 2025-01-15

**Authors:** Paige Conrad, Ranjeeta Brahmanand, Sudeep Yadav, Katrina R Gener, Alejandro Biglione

**Affiliations:** 1 Foundational Sciences, Nova Southeastern University Dr. Kiran C. Patel College of Osteopathic Medicine, Davie, USA; 2 Foundational Sciences, Nova Southeastern University Dr. Kiran C. Patel College of Osteopathic Medicine, Clearwater, USA; 3 Medicine, Univsersity of Chicago Pritzker School of Medicine, Chicago, USA; 4 Internal Medicine, B. P. Koirala Institute of Health Sciences, Dharan, NPL; 5 Internal Medicine, Wellington Regional Medical Center, Wellington, USA

**Keywords:** antibiotic-associated diarrhea, clostridium difficile, colitis, dyspnea, pleural effusion

## Abstract

*Clostridiodes difficile* is a gram-positive, spore-forming obligate anaerobe bacillus found in the intestines of healthy individuals without signs of disease. It may cause diarrhea after antibiotic use due to the eradication of the normal gut flora. Most cases resolve with proper treatment, but complications may arise. This case report is about a hospitalized patient who acquired a *C. difficile* infection after taking ceftriaxone, vancomycin, and linezolid for a cellulitis infection. During his hospitalization, the patient developed dyspnea with decreased breath sounds in the right lower lung lobe. A large pleural effusion in the right lung was observed on imaging, and analysis of the pleural fluid after thoracentesis revealed an exudative pleural effusion likely resulting from the *C. difficile* infection. The possible physiopathological mechanisms of pleural effusion in the setting of *C. difficile* infection are discussed.

## Introduction

*Clostridiodes difficile* is a gram-positive, spore-forming obligate anaerobe bacillus found in the intestines of healthy individuals without signs of disease [1]. *C. difficile* infection (CDI) commonly occurs after prolonged courses of antibiotics due to the eradication of normal gut flora and is more prevalent in individuals who are older in age, immunocompromised, or have had multiple hospitalizations [1-3]. In rare cases, extraintestinal manifestations, including a pleural effusion, may develop in CDI. The exact mechanism of pleural effusion-related CDI is not clearly understood, but inflammation of the bowel wall, leakage of albumin into the colonic lumen, and increased vascular permeability due to toxin-induced cytokines are believed to play a role in the pathogenesis [4]. This case report is about a hospitalized patient with *C. difficile *infection who developed a large pleural effusion during his hospitalization. The possible physiopathological mechanisms of pleural effusion in the setting of *C. difficile* infection are discussed. 

## Case presentation

A 40-year-old male patient with a past medical history of cellulitis presented to the hospital complaining of abdominal pain and diarrhea. The patient had received multiple courses of antibiotics, including ceftriaxone, vancomycin, and linezolid, for a cellulitis infection on his leg and subsequently began experiencing non-bloody, watery diarrhea for two months prior to presentation. The patient did not receive fluoroquinolone or clindamycin. The week before presenting to the hospital, he developed abdominal pain and began having episodes of watery diarrhea every 2 to 4 hours. He denied tobacco, alcohol, or recreational drug use. Upon presentation to the emergency room, his temperature was 36.5 ℃, blood pressure (BP) was 112/73 mmHg, pulse was 102 beats per minute, respiratory rate (RR) was 18 breaths per minute, and oxygen saturation was 94% on room air. On physical examination, the patient was awake, alert, and oriented to person, place, and time. He was in no acute cardiopulmonary distress. No jugular venous distention was observed, heart sounds were normal with regular rate and rhythm, and no murmurs or gallops were appreciated. The lungs were clear to auscultation. His abdomen was non-distended and diffusely tender to deep palpation. No rebound tenderness was observed and hyperactive bowel sounds were present on auscultation.

After recording the patient's history and physical exam findings, several differential diagnoses were considered, including viral gastroenteritis, inflammatory bowel disease, irritable bowel syndrome, foodborne illness, and a pancreatic neuroendocrine tumor such as a vasoactive intestinal polypeptide-producing tumor. 

The patient’s admission laboratory tests are shown (Tables 1, 2). A stool sample was collected and PCR testing for the presence of the *C. difficile* antigen and toxin was performed. The sample tested positive for the* C. difficile* toxin and antigen indicating the presence of a *C. difficile* infection. The patient was started on IV fluids and vancomycin 125 mg orally every 6 hours.

**Table 1:** Laboratory Values on Admission

	Initial Value	Normal Range
White blood cells	43.05 x10^3^/mcL	4.50 - 10.50 x 10^3^/mcL
Red blood cells	5.42 x10^6^/mcL	4.40 - 6.15 x 10^6^/mcL
Hemoglobin	15.6 gm/dL	14-18 gm/dL
Hematocrit	45.8 %	40.0-54.0%
Platelets	359 x 10^3^/mcL	150 - 450 x 10^3^/mcL
Mean corpuscular volume	84.5 Femtoliters	81.0 - 96.0 Femtoliters
Mean corpuscular hemoglobin	28.8 pg	27.0 - 34.0 pg
Mean corpuscular hemoglobin concentration	34.1 gm/dL	32-36 gm/dL
Red blood cell distribution width-standard deviation	41.2 Femtoliter	36.0-50.0 Femtoliter
Red blood cell distribution width-coefficient of variation	13.30 %	11.00 -14.50%
Neutrophil percentage	90.6 %	40-77%
Lymphocyte percentage	2.1%	24-44%
Monocyte percentage	4.9 %	0.0-15.0%
Eosinophil percentage	0.1 %	0.0-10.0%
Basophil percentage	0.4 %	0.0-2.0%
Immature granulocyte percentage	1.9 %	0.0-5.0%
Neutrophil number	39.01 x10^3^/mcL	1.80-7.70 x 10^3^/mcL
Lymphocyte number	0.9 x 10^3^/mcL	0.80-2.80 x 10^3^/mcL
Monocyte number	2.12 x 10^3^/mcL	0.20-1.00 x 10^3^/mcL
Eosinophil number	0.06 x 10^3^/mcL	0.00-0.60 x 10^3^/mcL
Basophil number	0.16 x 10^3^/mcL	0.00-0.30 x 10^3^/mcL
Immature granulocyte number	0.80 x 10^3^/mcL	0.00-0.50 x 10^3^/mcL

**Table 2:** Complete metabolic panel on admission

	Initial Value	Normal Range
Sodium	129 mmol/L	135-148 mmol/L
Potassium	4.4 mmol/L	3.6-5.2 mmol/L
Carbon dioxide	27.0 mEq/L	21-32 mEq/L
Chloride	95 mmol/L	95-110 mmol/L
Anion gap	11.4 mmol/L	5-15 mmol/L
Albumin	2.3 gm/dL	3.4-5.0 gm/dL
Blood urea nitrogen	10 mg/dL	7-18 mg/dL
Creatinine	1.35 mg/dL	0.70-1.30 mg/dL
Calcium	8.8 mg/dL	8.5-10.1 mg/dL
Glucose	169 mg/dL	74-106 mg/dL
Alanine transaminase	40 IU/dL	12-78 IU/dL
Aspartate aminotransferase	25 IU/L	15-37 IU/L
Estimated glomerular filtration rate of creatinine	68 mL/min/1.73m^2^	≥ 60 mL/min/1.73m^2^
Alkaline phosphatase	120 IU/L	45-117 IU/L
Total protein	6.4 gm/dL	6.4-8.2 gm/dL
Total bilirubin	0.8 mg/dL	0.0-1.0 mg/dL

**Figure 1:**
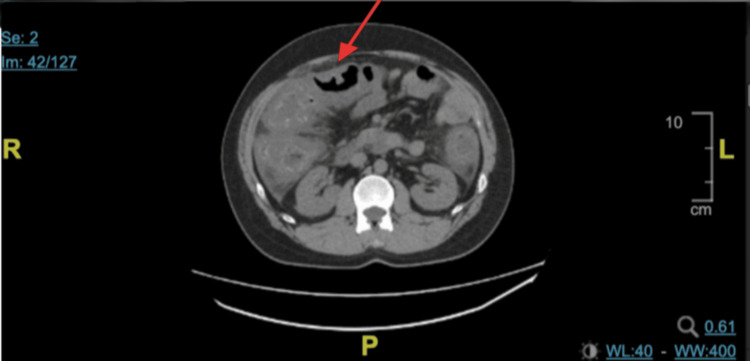
CT scan of the abdomen without contrast CT scan of the abdomen without contrast showed severely thickened and inflamed colonic wall consistent with pancolitis (red arrow). CT: computed tomography

A CT of the abdomen and pelvis without contrast showed colon wall thickening consistent with pancolitis (Figure 1).

**Figure 2:**
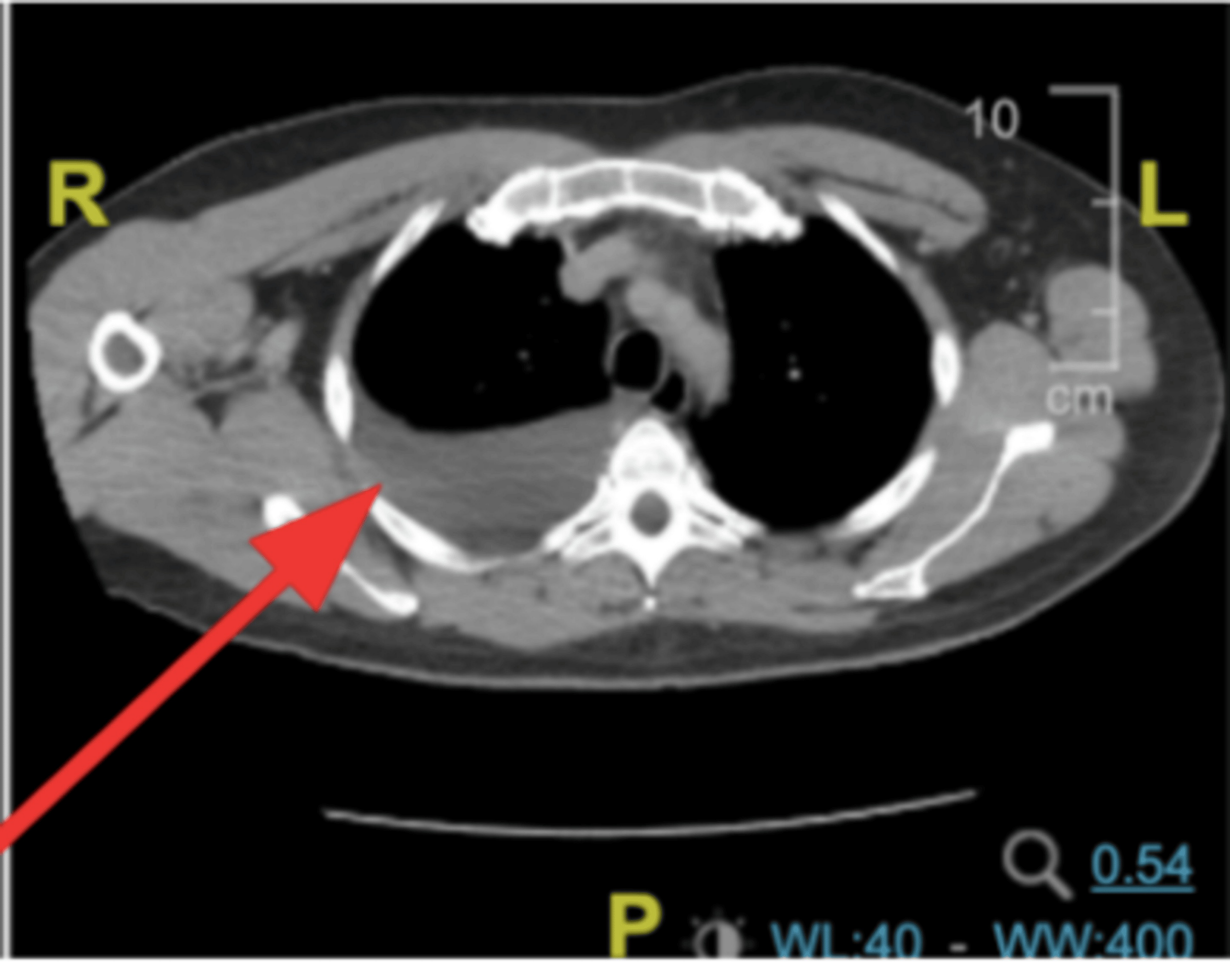
Axial CT of the abdomen with contrast CT scan of the abdomen with contrast showed a consolidation at the right lung base consistent with a large right pleural effusion (red arrow).

On Day 3 of his hospital stay, he complained of shortness of breath. His pulse was 106 beats per minute, respiratory rate was 20 breaths per minute, blood pressure was 116/73 mmHg, and oxygen saturation was 90% on room air. He was awake, alert, and oriented to person, place and time, and conversant. His cardiovascular exam showed normal heart sounds and a regular rate and rhythm. His chest exam showed dullness to percussion and decreased breath sounds at the right base. His abdomen was non-distended, mildly tender to deep palpation without rebound tenderness, and normal bowel sounds were auscultated. A chest x-ray showed a moderate right-sided pleural effusion, and a CT scan of the chest without contrast confirmed this finding (Figure 2). 

**Figure 3:**
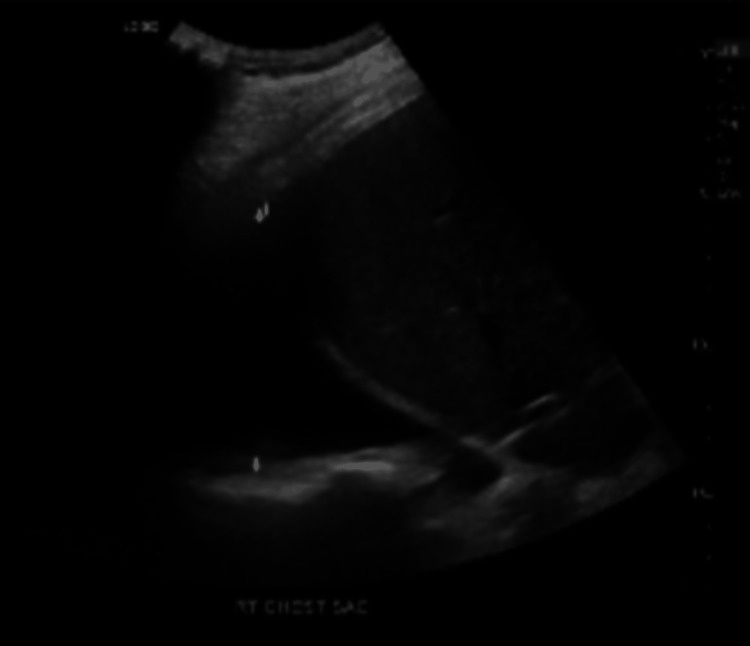
Ultrasound of the chest showed a large right pleural effusion

An ultrasound of the chest showed a large right pleural effusion (Figure 3).

He was started on oxygen via nasal cannula. A thoracentesis was performed with the removal of 800 ml of cloudy yellow fluid. Analysis of the fluid showed a pleural lactate dehydrogenase (LDH) to serum LDH ratio greater than 0.6 satisfying the criteria for an exudative pleural effusion according to Light's Criteria. The gram stain and cultures of the pleural fluid were negative, and cytology was negative for malignant cells (Table 3).

**Table 3:** Pleural fluid analysis LDH: Lactate Dehydrogenase

	Patient’s Fluid Analysis	Normal Range
Color	Yellow	Clear
Appearance	Hazy	Clear
White blood xells	1,035 cells/cm	< 1,000 cells/cm
Red blood cells	< 1,000 cells/cm	< 10,000 cells/cm
Segmented Neutrophils	90%	< 1%
Lymphocytes	2 %	20-25%
Pleural LDH	167 IU/L	< 50% of serum LDH
Serum LDH	269 IU/L	87-241 IU/L
Pleural LDH/Serum LDH	0.62	< 0.6
Pleural total protein	2.9 gm/dL	1-2 gm/dL
Serum total protein	6.2 gm/dL	6.4-8.2 gm/dL
Pleural protein/Serum protein	0.47	< 0.5

His dyspnea resolved after the thoracentesis, and the diarrhea gradually improved throughout his hospital stay with the regimen of oral vancomycin. The patient was discharged home and instructed to continue taking oral vancomycin 125 mg four times a day for an additional 5 days.

## Discussion

*Clostridium difficile* is a gram-positive, spore-forming obligate anaerobe bacillus that can be found in the intestines of healthy individuals without signs of disease [1]. It is the leading cause of hospital-acquired infections in the United States and is responsible for 15%-25% of all cases of diarrhea associated with antibiotic use [5-6]. The primary risk factor for *Clostridium difficile* infection is previous antibiotic use, particularly clindamycin, third and fourth-generation cephalosporins which our patient had previously taken, and fluoroquinolones [3]. Additionally, a weakened immune system, older age, proton pump inhibitor use, inflammatory bowel disease, recent gastrointestinal surgery, multiple hospitalizations or intensive care unit admissions, or residing in a long-term care facility are also risk factors for developing CDI [2-3].

The severity of the infection ranges from mild diarrhea, the presenting symptom in our patient, to severe fulminant colitis with toxic megacolon, sepsis, and colonic perforation [1,3,7]. Leukocytosis is frequently associated with CDI and is recognized as the leading cause of unexplained leukocytosis in hospitalized patients [7]. Our patient's leukocyte count was markedly elevated at presentation. Fever occurs in around 50% of cases, while bloody diarrhea is observed in a small minority [7]. Rapidly worsening leukocytosis, abdominal distension or pain, and an abrupt halt in diarrhea are considered poor prognostic indicators and have been anecdotally associated with fulminant colitis [7]. To date, only 14 cases of CDI presenting with ascites or pleural effusion have been documented, with a majority of these cases occurring in immunocompromised adults [4].

The gastrointestinal symptoms of CDI are a consequence of the eradication of normal gut flora from antibiotic use, and most cases resolve with appropriate treatment [1]. The exact mechanism of pleural effusion-related CDI is not clearly understood, but inflammation of the bowel wall leading to micro-perforations and infectious peritonitis, leakage of albumin into the colonic lumen, and increased vascular permeability due to toxin-induced cytokines are believed to play a role in the pathogenesis [4]. The clinical presentation of pleural effusions is largely dependent on the underlying etiology and the degree of inflammation between the parietal and visceral pleural layers. However, pleural effusions most frequently present as dyspnea in addition to a generalized painful pressure-like feeling in the chest or a dry cough [8]. 

A cross-sectional study found a positive correlation between fecal *C. difficile* toxin levels and disease severity with extremely high toxin levels potentially indicating worse outcomes and higher short-term mortality in these patients [9]. The prognosis for most patients with CDI is generally positive. However, adverse events, such as colectomy or death, were observed in up to 17.3% of the inpatients with hospital-acquired CDI at the University of Pittsburgh over a two-year period, and a 30-day mortality rate of 6.9% was found in a study of patients in 20 hospitals [7]. An all-cause mortality rate of 50% is seen in individuals who need a colectomy [7].

Current treatment recommendations emphasize the use of 125 mg of oral vancomycin four times a day or 200 mg of oral fidaxomicin every 12 hours alone for patients identified as being at risk for severe disease based on various clinical criteria. For patients with milder CDI, 500 mg metronidazole three times per day for ten days or 125 mg vancomycin four times a day for ten days is the recommended treatment [10]. As this was our patient's first infection with *C. difficile* and no signs of sepsis or toxic megacolon were observed, 125 mg oral vancomycin four times per day for 10 days was prescribed in this case. Fecal microbiota transplantation has been shown to be more effective than vancomycin in the treatment of recurrent CDI, but the potential dangers of short and long-term donor-derived infection, especially in immunocompromised individuals, has prevented the universal application of this therapy [7]. 

Thoracentesis is a diagnostic and therapeutic procedure for complications like pleural effusion, and it is always advised when the effusion's etiology is unknown.[8]. In cases of large pleural effusions causing significant respiratory distress or recurrent pleural effusions, placement of a pleural drain may be necessary [8]. One of the most concerning features of CDI is its high relapse rate, which ranges from 10-35% after an initial episode, approximately 40% after the first recurrence, and 60-100% after two or more recurrences. Advanced age, immunosuppression, and continued antibiotic use are strong risk factors for recurrent CDI [7]. 

## Conclusions

*Clostridium difficile* may cause diarrhea after antibiotic use due to the eradication of the normal gut flora. Large pleural effusions requiring thoracentesis seldom develop. This case report about a patient with a *C. difficile* infection who developed a large pleural effusion highlights this rare complication. After receiving a thoracentesis and taking oral vancomycin for five days, the patient reported a significant improvement in symptoms. He was discharged home and instructed to continue taking oral vancomycin 125 mg four times a day for an additional five days. Although the exact physiopathological mechanisms of the development of pleural effusions in patients with *C. difficile* are not clearly understood, protein-losing enteropathy due to inflammation of the bowel wall and leakage of albumin into the colonic lumen is a potential mechanism. This complication should be kept in mind in patients with *C. difficile *infection who develop dyspnea and pleural effusion. Thoracentesis may be required to clarify the diagnosis and to alleviate symptoms. To prevent the occurrence of *C. difficile* infection, careful prescription and adherence to antibiotic regimens should be practiced. Additionally, proper hand hygiene measures and the use of personal protective equipment, such as gowns and gloves, should be implemented to prevent the spread of infection, particularly in healthcare environments. 
